# A cohort study of *Plasmodium falciparum *infection dynamics in Western Kenya Highlands

**DOI:** 10.1186/1471-2334-10-283

**Published:** 2010-09-24

**Authors:** Frederick N Baliraine, Yaw A Afrane, Dolphine A Amenya, Mariangela Bonizzoni, Anne M Vardo-Zalik, David M Menge, Andrew K Githeko, Guiyun Yan

**Affiliations:** 1 University of California - San Francisco, Department of Medicine, Division of Infectious Diseases, P.O. Box 0811, San Francisco, CA 94143-0811, USA; 2 Center for Vector Biology and Control Research, Kenya Medical Research Institute, Kisumu, Kenya; 3 Liverpool School of Tropical Medicine, Liverpool, UK; 4 University of California - Irvine, Irvine, CA 92697, USA; 5 Department of Biology, Pennsylvania State University, York, PA, USA; 6 University of Minnesota, Minneapolis, MN, USA

## Abstract

**Background:**

The Kenyan highlands were malaria-free before the 1910s, but a series of malaria epidemics have occurred in the highlands of western Kenya since the 1980s. Longitudinal studies of the genetic structure, complexity, infection dynamics, and duration of naturally acquired *Plasmodium falciparum *infections are needed to facilitate a comprehensive understanding of malaria epidemiology in the complex Kenyan highland eco-epidemiological systems where malaria recently expanded, as well as the evaluation of control measures.

**Methods:**

We followed a cohort of 246 children residing in 3 villages at altitudes 1430 - 1580 m in western Kenya. Monthly parasitological surveys were undertaken for one year, yielding 866 *P. falciparum *isolates that were analyzed using 10 microsatellite markers.

**Results:**

Infection complexity and genetic diversity were high (H_E _= 0.787-0.816), with ≥83% of infections harboring more than one parasite clone. Diversity remained high even during the low malaria transmission season. There was no significant difference between levels of genetic diversity and population structure between high and low transmission seasons. Infection turn-over rate was high, with the average infection duration of single parasite genotypes being 1.11 months, and the longest genotype persistence was 3 months.

**Conclusions:**

These data demonstrate that despite the relatively recent spread of malaria to the highlands, parasite populations seem to have stabilized with no evidence of bottlenecks between seasons, while the ability of residents to clear or control infections indicates presence of effective anti-plasmodial immune mechanisms.

## Background

*Plasmodium falciparum *malaria is presently among the most fatal diseases in the East African highlands, where an estimated 34 million people remain at risk [1]. Unlike the endemic lowlands, these relatively cool high-elevation areas were basically malaria-free before the 1910s. Sporadic malaria epidemics occurred from the 1920s to the 1950s, but a series of highly fatal epidemics have occurred in the highlands of western Kenya since the 1980s, the most recent occurring during 2004 [2]. Unlike the lowlands where malaria has been endemic for generations, populations in highland areas that are less exposed to malaria parasites are expected to be less immune and hence more vulnerable to epidemics. It is probable, however, that successive waves of infection may have generated some level of immunity among the highland residents. Immunity to malaria comes gradually, consequent to repeated or persistent infection for years, during which period an individual develops immune responses to most parasite variants circulating in a particular area. The fact that the diversity of malaria parasites is a sequel of both clonal antigenic variation and allelic polymorphism means that the period of infection persistence is an important parameter [3]. Moreover, acquisition of anti-plasmodial immunity can affect infection dynamics by shortening infection persistence [4].

In a previous study, we detected plasmodial infections in one highland area of Kenya with some individuals being more frequently parasitemic than others, but we did not know whether this was due to infection persistence by one or more strains or due to re-infections [5]. Obviously, estimates of parasite burden based solely on parasite detection provide only a rudimentary measure, since prevalence is a consequence of various factors, such as infection persistence or the rate of relapse of latent infections, the incidence of new infections, and the rate of infection clearance from the circulation [6]. As such, single-time-point sampling as occurs in cross-sectional studies may not give a comprehensive epidemiological picture [7]. A longitudinal tracking of parasites within individuals would therefore provide better insight into the role of such factors in infection dynamics, while at the same time providing a surrogate measure of population immunity [3,4]. Studies of the duration of plasmodial infections in natural populations are lacking in the East African highlands. Moreover, without parasite typing data, it is impossible to reliably estimate the duration of infections among residents of areas with ongoing transmission [8]. The use of multiple genetic markers helps prevent the overestimation of clonal duration by minimizing the chance of misclassifying new infections as persistent, as the probability of different plasmodial strains having identical genotypes at multiple markers is reduced [9].

Malaria parasite populations in endemic Africa are highly diverse, varying both genetically and phenotypically (virulence, drug resistance, and transmissibility) [10]. Available data show that there is a direct correlation between the number of genotypes per person and transmission intensity [11,12]. Many recent cross-sectional studies have drawn attention to the relationship between the multiplicity of infection (MOI), transmission intensity, and genetic structure. While these studies have established that MOI is negatively associated with disease severity, they also provide another reliable surrogate measure of transmission intensity and population immunity [7,13,14]. However, like infection duration studies, longitudinal studies of infection complexity and dynamics are lacking in the East African highlands.

In this paper, we report a longitudinal study on malaria parasite population genetic diversity and infection dynamics in the western Kenya highlands based on a cohort of school children. We utilize highly polymorphic microsatellite markers to compare infection complexity, genetic diversity, and population structure between transmission seasons to determine if a reduction in transmission has an effect on parasite population structure in the highlands. Understanding malaria parasite population genetic structure and infection dynamics may be useful in the assessment of malaria control strategies [15]. In addition, we determine how long individual malaria parasite genotypes persist in humans. Infections that last longer have higher transmission potential [16] and also indicate relatively low levels of immunity, while quickly cleared infections indicate high levels of population immunity [4,13].

## Methods

### Study area and population

This study took advantage of the availability of samples from our previous study [5]. The study was carried out in 3 villages: Iguhu, Makhoko and Sigalagala at altitude 1430-1580 m in Kakamega district, Western Kenya. The area has two rainy seasons, averaging 1800 mm rainfall annually. The long rainy season occurs between mid-March to May, while September-October is the short rainy season. Malaria prevalence peaks in this area usually lag 1-2 months after the rains [17]. Over the past few decades, the area has undergone significant land-use changes with massive deforestation, inducing micro-climatic changes that enhance survivorship and reproduction of *Anopheles gambiae *mosquitoes, the main malaria vector species in this area [18]. Daily temperatures range between 13.8 - 28°C. The entomological inoculation rate (EIR) at Iguhu was 16.6 during 2003-2004 [19], while it was 0.05 and 0.04 at Makhokho and Sigalagala respectively during 1999-2000 [20]. Other details of the study area have been previously reported [21]. The DNA analyzed in this study was obtained from a cohort of 246 randomly selected apparently healthy primary school children (84 from Iguhu, 81 from Makhokho and 81 from Sigalagala). A questionnaire was used to capture demographic data and treatment history. The children (5-17 years) were followed from January to December 2006 with monthly parasitological surveys, after obtaining their assent and informed consent from parents or guardians. Blood smear results were immediately reported to the study participants and their parents or guardians. However, since the Kenya Ministry of Health guidelines did not advise treatment of asymptomatic persons, parasitemic individuals were advised to seek anti-malarial treatment at the nearby Iguhu Health Center if they subsequently developed fever or any other signs of malaria, such as chills, headache, muscle pain, nausea, or vomiting. The study was approved by Kenya Medical Research Institute Ethical Review Committee and the Institutional Review Board of the University of California, Irvine.

### Microsatellite genotyping

Parasite DNA was extracted from the study subjects' blood samples preserved on Whatman 3 MM filter papers, using saponin/chelex-100, and infections were diagnosed by nested PCR amplification of the species-specific small-subunit ribosomal RNA gene, as previously reported [22]. The PCR products were run in ethidium bromide-stained 2% agarose gels, and visualized under ultraviolet light. Negative PCR samples were re-amplified, and infections were declared negative only after repeated DNA extraction and re-amplification for cases where the initial DNA extract was negative. Genomic DNA from 866 *P. falciparum *PCR-positive samples was then genotyped using 10 microsatellite markers (TA1, TA42, TA81, TA87, TA109, ARA2, 2490, Polyα, Pfpk2, Pfg377) [23], on LI-Cor 4300 automated genetic analyzer, using previously reported protocols [24].

Since blood-stage malaria parasites are haplotypic, the presence of more than one allele at any one locus was considered diagnostic of a mixed infection. Only minor alleles with peaks ≥33% the height of the predominant allele were considered in case of mixed-clone infections [25]. Where mixed infections occurred, the predominant allele was used in estimates of allele frequencies and population structure [15,25].

### Analysis of genetic diversity and population structure

We examined the difference in genetic diversity between low and high malaria transmission seasons. To avoid compounding effects of repeated sampling, the low transmission season was represented by samples collected in April, while the high transmission season was represented by the June samples; based on a consideration of both the usual malaria transmission patterns in the area [17], and our monthly malaria prevalence data. Genetic diversity of *P. falciparum *infections was measured by the number of observed (Na) and effective (*ne*) alleles per locus, Nei's unbiased genetic diversity (H_E_), Nei genetic distance (*D*), and Shannon's index (I), using GenAlEX6.1 software [26]. A paired t-test was used to compare differences in mean H_E _values obtained at each locus during the low and high transmission seasons. Because no genetic differentiation was observed among the three villages (*D *= 0; respective H_E _values for Iguhu, Makhokho and Sigalagala were: 0.82, 0.77 and 0.79 for the low and 0.80, 0.79 and 0.81 for the high transmission season), samples from the three sites were pooled and analyzed as the same population. Proportions of mixed-genotype infections between seasons were compared using Fisher's exact test. Hierarchical analysis of molecular variance (AMOVA) was performed to determine the genetic variation between the low and high transmission seasons using ARLEQUIN [27].

Factors such as close physical linkage among loci, inbreeding/selfing, genetic drift, or the Wahlund effect may cause linkage disequilibrium (LD) [28]. Estimates of LD can thus give important clues about population structure and history. Pair-wise LD between markers was examined for the low and high transmission seasons, using GENEPOP3.4 [29].

### Effective population size and genetic bottleneck analysis

The effective population sizes (Ne) in the low and high transmission seasons were calculated from the observed heterozygosity, assuming a microsatellite mutation rate of 1.59 × 10^-4 ^for *P. falciparum*, as previously described [25]. Evidence for severe reduction in Ne (genetic bottleneck) between the two seasons was further examined by the Wilcoxon Signed-Rank test, under three models: the infinite alleles model (IAM), stepwise mutation model (SMM) and two-phase model (TPM), using BOTTLENECK1.2.02 [30]. Evidence for allele frequency distortion which occurs at neutral loci during bottlenecks was also assessed using the "mode-shift" indicator [31].

### Differentiating between new and pre-existing infections and estimates of infection duration

To differentiate between new and pre-existing infections and to estimate durations of infecting parasite genotypes, we followed, with modification, the method of Franks and colleagues [3], which utilized the analysis of alleles at multiple loci. Briefly, we first identified and ranked the most polymorphic loci by obtaining heterozygosity data for the 10 microsatellites across all samples. The most polymorphic loci, with H_E _≥ 0.8 (Polyα, TA109, TA87, TA1, Pfpk2, TA81 and ARA2 in descending order) were chosen for this analysis. The January samples were used as the reference baseline genotype. If the parasite sample in the subsequent month showed distinct alleles in the most polymorphic locus (Polyα), we examined the next most polymorphic locus (TA109) to determine whether new alleles were present at this locus as well, until all 7 loci were examined with reference to the baseline. The prevalence of new infections was estimated sequentially on a month-by-month basis. Thus, if a new allele absent in a previous month was observed, a new infection was counted. On the other hand, if each of the loci showed at least one identical allele to the baseline sample, the initial infection was considered persistent. However, PCR may fail to amplify a clone when parasitemia is low [32] or when parasites are sequestrated [7,9]. To account for this possibility, we accommodated one missing data point between consecutive months, such that if, for example, the initial allele was present in the second month but not detected again until the fourth month, we still considered it a persistent infection. The duration of a parasite genotype was then calculated as the summation of the number of consecutive months from the initial infection to last month of persistence.

The use of single polymorphic markers in determining malaria parasite genotype persistence has also been reported [33]. Because the ten microsatellite markers vary in polymorphism, the available dataset offered us an opportunity to evaluate the effects of marker polymorphism on the estimated infection durations. We determined genotype durations using each of the 10 microsatellite markers, and then employed the Kaplan-Meier survival estimator to compare the overall infection durations. Mean infection duration estimates among locus pairs were then compared using the Tukey-Kramer HSD test (JMP 5.1, SAS Institute).

### Estimates of incidence rates

Incidence rates were estimated based on new infections among individuals who were *P. falciparum*-negative at the beginning of the study (n = 139); i.e. considering only the first infection detected in each of these individuals during the year. We also determined the proportion of infected individuals (n = 157) that acquired additional infections during the 12-months study period.

## Results

246 subjects were enrolled in the study, but 19 were lost to follow-up over the first 11 months, with an additional 170 subjects lost to follow-up during final month of the study, due to the Christmas recess. 53 of the 246 subjects in the cohort reported developing clinical malaria, with antimalarial treatment on a total of 77 occasions. 63.6% (49/77) of the cases were self-medicated, and the remaining (36.4%) were treated by clinicians. 92.2% (71/77) of the cases were treated with sulfadoxine-pyrimethamine, and 3.9% (3/77) with amodiaquine or quinine. Nearly half (43.4%) of 53 subjects who reportedly had clinical signs of malaria and received treatment at some point during the study were parasite-negative at the times of our sampling, throughout the year. Overall, 30 of these 53 subjects contributed 41 parasite-positive blood samples during the study period; 23 of these samples were obtained at least two months before or after treatment, while 18 were obtained within one month post-treatment, of which 16 of these 18 were new infections. Overall, a total of 866 samples were genotyped during the 12-months study period.

### Microsatellite diversity and analysis of molecular variance (AMOVA)

**Figure 1:**
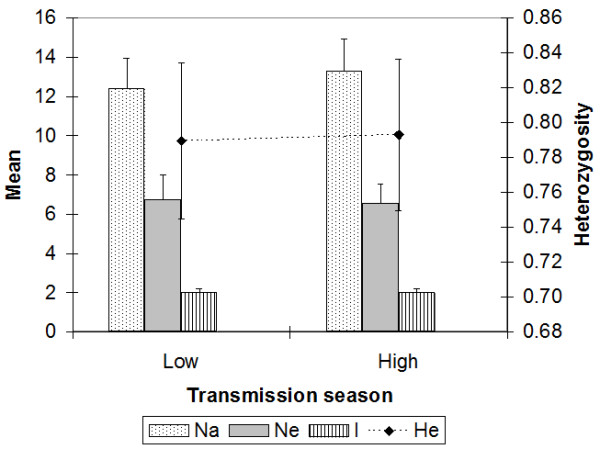
**Microsatellite allelic patterns across transmission seasons**. No significant difference in diversity was observed between the low and high malaria transmission seasons. Whiskers represent standard error values.

The microsatellite markers were highly polymorphic, with monthly mean number of alleles per locus being 12.2, ranging from 8 (Pfg377) to 27 (Polyα). Genetic diversity was high throughout the year (H_E _range: 0.787 - 0.816), with monthly number of alleles (Na) substantially higher than the number of effective alleles (*ne*) [range = 3 - 27 vs 1.5 - 17.6, respectively; mean Na - *ne *= 5.7, 95% CI = 5.2 - 6.1; t = 23.2 df = 119, *P *< 0.001]. Variability data for the low versus high malaria transmission seasons were respectively as follows: Na = 12.4 vs 13.2; *ne *= 6.75 vs 6.52; He = 0.789 vs 0.793, and I = 2.0 for both seasons (Figure 1). Parasite population unbiased genetic diversities for the low versus high transmission seasons were both high and exhibited no statistical difference (H_E _= 0.803 vs 0.803; 95% CI = -0.016 - 0.016, t = 0, df = 9, *P *= 1.0). AMOVA revealed no significant difference in genetic structure between the low and high transmission seasons (Table 1).

**Table 1:** Analysis of molecular variance (AMOVA) among *Plasmodium falciparum *populations during the low and high transmission seasons in a highland area of western Kenya.

Source of variation	df	SS	Variance components	Percent variation	*P *value
Between seasons	1	5.23	0.018	0.45	0.058
Within seasons	147	550.31	4.014	99.55	
Total	148	555.55	4.032	100	

### Linkage disequilibrium

Only three and one out of 45 possible pair-wise tests showed significant LD (*P *< 0.05) in the low and high transmission seasons, respectively, with no significant association involving loci on the same chromosome. Moreover, no locus pair remained in LD after applying both the highly conservative Bonferroni correction and the less conservative Benjamini-Yekutieli false discovery rate (B-Y FDR) procedure for multiple comparisons [34,35].

### Effective population size and bottleneck analysis

Based on the formulae and assumptions reported by Anderson and colleagues [25], effective population size (Ne) was high during both seasons; Ne with 95% confidence intervals were: 9037 (3883, 20585) and 8727 (3750, 19880) under IAM, and 46193 (19850, 105224) and 39808 (17107, 90679) under SMM for the low and high transmission seasons, respectively. Bottleneck analyses using the Wilcoxon Signed-Rank test under all known microsatellite mutation models gave no significant result, except for a marginally significant bottleneck under IAM for the low transmission season (Table 2). Overall, alleles showed a normal, L-shaped distribution across seasons, suggesting no significant seasonal decline in effective population size in this highland area.

**Table 2:** *P *values for the bottleneck analysis of *Plasmodium falciparum *populations during the low and high malaria transmission seasons in a highland area of western Kenya.

Model^§^ Season	IAM	SMM	TPM	Mode-shift
Low transmission (n = 64)	0.04199*	0.99902	0.72168	Normal
High transmission (n = 85)	0.05273	1.00000	0.83887	Normal

^§ ^The Wilcoxon test was used to test for heterozygosity excess under infinite allele (IAM), stepwise mutation (SMM) and two-phase (TPM) models. *P *values are indicated under each model type. Parameters for TPM were: variance = 30; proportion of SMM = 70%. Estimates were based on 1,000 replications.

n = number of samples tested.

* Indicates statistical significance at *P *< 0.05.

### Monthly infection complexity and stability of the minimum number of clones

**Figure 2:**
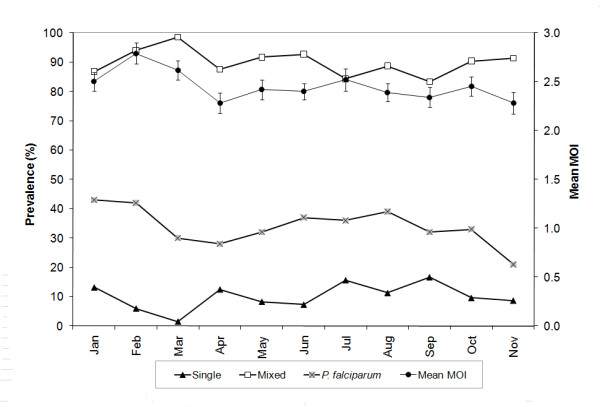
**Monthly prevalence of *Plasmodium falciparum *infections, indicating the proportions of single and mixed infections and mean multiplicity of infection (MOI)**. Prevalence of mixed infections was high, with mean MOI consistently above 2.0 throughout the year. Whiskers represent standard error values. December data were excluded because of insufficient sample size (n = 11; but all were mixed infections).

Data for December were not used for monthly infection complexity analysis because of a small number of infected samples (n = 11; all multi-clonal). The prevalence of mixed-clone infections was high (83.3 - 98.5%), while the prevalence of single infections was generally low (< 17%; Figure 2). There was a significant difference in infection prevalence during the low (28%) and high (37%) transmission seasons (*P *= 0.039), but no difference was observed in infection complexity between the two seasons (*P *= 0.641). The average number of *P. falciparum *clones per infection for the entire study period was 2.5 (range 1- 6).

### Incidence rates and infection persistence

The estimated plasmodial infection incidence rate was 669 per 1,000 person years. The reinfection or superinfection rate was high (86.6%). The multilocus estimation showed the average duration of individual parasite genotypes to be 1.11 months, with the majority (92.2%) of infecting genotypes persisting for only one month (range 1-3 months). Considering infections from individuals who fell sick and got malaria treatment at some point during the year versus those who never got treatment at all, the proportion of infections lasting more than one month was higher among individuals who received treatment than among those who were asymptomatic throughout the test period and never got treated (20% vs 4.7%, *P *= 0.01). There was a wide variation in infection durations estimated by individual microsatellite markers. The less polymorphic loci gave longer genotype persistence estimates. The highly polymorphic markers (e.g. Polyα and TA109) yielded statistically similar mean infection duration estimates to the multilocus estimation (Table 3).

**Table 3:** Comparison of infection duration estimates among individual microsatellite markers and the multilocus approach that involves 7 loci with H_E _> 8.0

Markers*	Mean Duration (Months)	Range (Months)					H_E_
TA42	3.96	1-12	A				0.457
2490	2.87	1-12		B			0.737
Pfg377	2.74	1-12		B			0.660
ARA2	2.31	1-11		B	C		0.857
TA81	2.23	1-11		B	C		0.857
TA1	2.16	1-10		B	C		0.892
TA87	1.87	1-11			C		0.901
PfPK2	1.85	1-10			C	D	0.860
Polyα	1.65	1-10			C	D	0.940
TA109	1.64	1-10			C	D	0.903
Multilocus - 7 markers (C)	1.11	1-3				D	

*Markers not sharing the same letter in a column are significantly different (*P *< 0.05) using the Tukey-Kramer honestly significant differences (HSD) test.

## Discussion

The dynamic nature of plasmodial infections renders longitudinal studies the most desirable for a comprehensive understanding of malaria epidemiology [7], more so in the complex highland eco-epidemiological systems where malaria recently expanded [2]. In this study, we employed a panel of 10 highly polymorphic microsatellite markers to determine the seasonal population structure, infection duration, complexity and dynamics throughout the year in one highland area of western Kenya.

Samples from the three villages were analyzed together, since the villages are near to each other (within a 5 km radius) and there were no differences in genetic diversity observed between the low and high transmission seasons, contrary to what would be expected based on the substantial differences in EIR among the sites. A number of possible explanations may account for this observation. First the EIR data cited were obtained in different years; perhaps a clearer EIR picture could have been obtained with data obtained during the study period. Second, vector sampling for EIR estimates may have not represented all the areas where the children resided [20]. Third, human travel may have facilitated parasite introduction and high gene flow across the villages [2,24,36], also helped by both the ready availability of the asymptomatic, untreated malaria reservoir and the notably high efficiency of malaria transmission by the vectors in the study area [20]. In fact, earlier studies carried out in the study area found no correlation between EIR and parasite prevalence or density [20], and similar conclusions were derived from studies carried out in Tanzania [37].

The high infection incidence rate, high parasite population genetic diversity, together with the high level of among individual variability depicted by AMOVA suggests that there is substantial heterogeneity in *P. falciparum *haplotype composition within this highland area. Moreover, the absence of genotypic linkage disequilibrium indicates that factors such as inbreeding/selfing, genetic drift, or the Wahlund effect were not sufficiently significant to cause changes in parasite genetic structure [28]. The recent introduction of artemisinin-based combination therapies (ACTs) has made a significant impact on the clinical malaria burden in much of sub-Saharan Africa, based on hospital data [38]. In Kenya, ACTs were rolled out from July 2006 [39]. Three recent studies in Kenya show substantial reductions in the malaria burden in some areas, but hospital data show that there still exist many areas where malaria is not declining [38]. Interim data from studies of malaria in western Kenya before 2005 and 1-2 years after introduction of ACTs also show a mixed pattern, with some high transmission lowland areas showing a sharp decrease in malaria prevalence during early 2007, followed by a sharp increase in the late 2007-2008 [40]. We have also noted a reduction of malaria prevalence in our study sites in 2007-2008, and recovery of malaria prevalence since 2009 (Y. Afrane, unpublished data). Consistent with our findings, no significant differences in population structure between the lowland and highland sites were observed over the years, suggesting that the current malaria control efforts have not caused a major change in parasite population structure [40]. African mosquitoes are highly efficient malaria transmitters [20,41], and while ACTs kill gametocytes, the infective stage for mosquitoes, they may not have a quick impact on malaria transmission in areas where a minority of infected individuals fall sick and seek treatment [38], necessitating a consideration of treating the infected, asymptomatic group if malaria is to be eliminated. Our study focused on asymptomatic subjects, a significant majority of whom received no antimalarial drug treatment. Broadly, considering that only about 25% of plasmodial inoculations in Africa result in clinical disease [41], and because current treatment policies focus on clinically sick people, with most estimates of malaria burden based on hospital clinical data, the contribution of parasites from the asymptomatic reservoir both to genetic structure and estimates of malaria burden may often be unnoticed.

More than 83% of the asymptomatic infections were multi-clonal throughout the 12-months study period. Similar estimates were obtained from earlier cross-sectional studies of symptomatic patients in the highlands of Kakamega and Kisii, and the low lands of Kombebwa; all in western Kenya [24]. Various epidemiological studies have established that there is a correlation between the prevalence of multiple-genotype infections and malaria transmission intensity [25,42]. While seasonal fluctuations in MOI are common in low transmission areas such as eastern Sudan (EIR <1) [16], we found no difference in MOI between the low and high transmission seasons. This is not surprising because firstly, our study area is transected by a major river at the valley bottom, which provides a steady breeding ground for malaria vectors throughout the year [5,43]. Secondly, a significant majority of the infections were asymptomatic, hence not treated, thereby having a greater opportunity for recombination than if they were treated with effective drugs such as ACTs. Our results are consistent with those from elsewhere, which show that in areas of relatively high transmission, the number of clones in asymptomatic infections tend to be consistent over long periods of time, despite fluctuations in malaria transmission intensity [44].

Application of molecular genotyping to longitudinally-collected parasite samples provides high utility in studying infection dynamics, as it enable distinction between pre-existing and newly acquired infections. Without genotyping, we previously found detectable *P. falciparum *parasitemias among individuals in this area for up to 12 months [5]. The present multi-locus typing analysis, however, shows the longest single genotype duration being only 3 months, sharply contrasting with studies in Ghana where parasite genotypes persisted for at least 10 months [3] and for over 12 months in Sudan [16]. The significantly longer infection duration estimates obtained with individual loci (up to 10-12 months) compared to multiple loci is consistent with the view that multi-locus analysis helps prevent over-estimation of infection duration [45]. Moreover, we allowed for the dynamic nature of *P. falciparum *parasitemia [7,46] by accommodating one missing data point in our duration estimates. The high prevalence of *P. falciparum *observed in our study area thus appears to be a sequel of more frequent clearance and acquisition of infections, rather than their protracted persistence.

It is difficult to determine the impact of antimalarial drug treatment on infection clearance in our study. First, the study was carried out in 2006, when resistance to the most commonly used drug, sulfadoxine-pyrimethamine, was supposed to be high. Majority of samples obtained within one month after treatment were parasite-negative, with genotyping results showing only 2 out of 18 positive samples obtained within one month of treatment being recrudescent; the rest were new infections. Second, nearly half (43.4%) of the subjects who reportedly had clinical signs of malaria and took treatment at some point during the study period were parasite-negative at the times of our sampling, throughout the year; raising questions whether they were true malaria cases that adequately responded to treatment and never got re-infected. Because there are many causes of fever and other malaria-like symptoms, cases of presumptive treatment of fevers with antimalarial drugs abound in Africa, with clinicians ignoring laboratory results in many cases [47]. Overall, however, although the majority were parasite-negative one month after treatment, the was a higher proportion of persistent infections among the group of individuals who reportedly developed clinical signs of malaria and got treated than among those who were asymptomatic throughout the study period. It is probable that a high proportion of persistent infections among the group that ultimately succumbed to clinical disease was caused by relatively lower immunity levels and hence relatively lower efficiency at clearing infections compared to the untreated group [48]. Nevertheless, exclusion of all individuals that reportedly took antimalarial drugs at any given time of the year did not change the overall estimated duration of asymptomatic parasitemias in our study area.

One limitation of our study was that fever was not measured; hence we cannot rule out the possibility that some of the parasitemias were associated with clinical malaria. However, besides fever, clinical malaria is often associated with other symptoms, such as shivering, headache, muscle weakness, joint pain and vomiting. None of these symptoms were observed at the times of sampling, and the majority of the children did not report to have fallen sick afterwards or taken any antimalarial drug throughout the study period.

The rapid infection turnover observed in our study area is typical of high transmission areas, and the observed longest single genotype duration is within the expected range (50 - 150 days) for areas of high transmission [49,50]. These data indicate that a substantial number of individuals tested in this area may have developed sufficient immunity to deal with peripheral parasitemia, hence the ability to clear infections faster [4,7,51].

In a population having a constant effective size over the recent past, there is an equal probability that loci show excess or deficit of heterozygosity [30]. Overall, no heterozygosity excess was observed, implying there were no bottlenecks in the *P. falciparum *population during the low and high malaria transmission seasons in this highland area [25,30]. The high effective population sizes observed in this area are important, because the bigger the Ne, the better prepared will the population be to resist genetic drift and to effectively adapt to adverse environmental or other changes or pressures [52].

The observed high re-infection rates call for unrelenting malaria control efforts, which should not ignore the asymptomatic sub-population, if malaria is to be ultimately eliminated in this area. The existence of parasite populations that are largely sheltered within the asymptomatic sub-population in this highland area, together with a high prevalence of mixed infections pose a challenge to control efforts. First, asymptomatic parasitemias are often so low that they often escape detection by microscopy, the widely used tool for malaria diagnosis [5]. Second, multi-clonal infections avail greater potential for enhanced parasite recombination and clonal expansion, produce more gametocytes [16] and some studies have found them to be more infectious to mosquitoes than single-clone infections [53,54]. These parasitemias may provide a substantial gametocyte reservoir both for continual infection and as a precursor for the next transmission season or epidemics when mosquitoes become more abundant [2]. Moreover, recent anti-malarial drug efficacy studies in 7 areas in Uganda with widely varying malaria transmission intensities (EIRs 7 - 1564) have demonstrated that high MOIs are associated with a greater risk of treatment failure [55]. Furthermore, malaria immunity is strain-specific, hence high MOIs pose an obstacle to the development of effective malaria vaccines [56]. Third, we observed a high effective population size (Ne) and a normal mode shift, with lower *ne *than Na suggesting the presence of adaptive, low-frequency alleles [25,52,57], meaning that *P. falciparum *populations in this area may be having a high potential for responding to environmental changes and pressures imposed by control measures.

## Conclusions

Taken together, these data demonstrate that despite the relatively recent spread of malaria to the highlands, parasite populations seem to have attained a high effective population size, while the ability of the majority of residents to clear or control infections indicates presence of effective anti-plasmodial immune mechanisms. Asymptomatic parasitemias may be playing a large role in maintaining the parasite burden in this area; they are not presently targeted for treatment. Since the high parasite population size, with high multi-clonality give a biological advantage to the parasites for their survival and spread [16,54], the use of efficacious drugs with gametocyticidal activity, such as ACTs will go a long way in curtailing the apparent parasites' biological advantages and prevent possible epidemics. A similar strategy was successfully employed to block transmission and prevent imminent epidemics on the Thai-Burmese border [58], in Eritrea, Zanzibar, and KwaZulu-Natal in South Africa [59]. However, given the above factors, it will be necessary not only to deploy, but also to closely and continuously monitor the progressive effectiveness of all control interventions.

## Competing interests

None of the authors has a commercial or other association that might pose a conflict of interest in this work.

## Authors' contributions

FNB: Carried out the molecular genetic studies, performed the statistical analyses, data interpretation and drafted the manuscript. YA: Conducted sample collection and helped with writing the manuscript. DAA: helped in writing of the manuscript. MB: Participated in data analysis and writing of the manuscript. AMV-Z: Participated in data analysis and writing of the manuscript. DMM: Participated in writing of the manuscript. AKG: Participated in the design of the study, facilitated, and conducted field sample collection. GY: Conceived the study, acquired funding, and participated in sample collection and in manuscript preparation. All authors read and approved the final manuscript.

## Financial support

This study was funded by grants R01 AI050243, D43 TW001505, and R03 TW007360 from the National Institutes of Health of the USA.

## Note

Our dear colleague, Dr David Maina Menge passed away on Saturday 29th May 2010, when the manuscript was still under revision. We dedicate this paper to him.

## Pre-publication history

The pre-publication history for this paper can be accessed here:

http://www.biomedcentral.com/1471-2334/10/283/prepub
